# Unilateral Stage 1A Macular Hole Secondary to Low-Energy Nd:YAG Peripheral Iridotomy

**DOI:** 10.7759/cureus.12603

**Published:** 2021-01-10

**Authors:** Jonathan C Tsui, Steven J Marks

**Affiliations:** 1 Ophthalmology, Geisinger Medical Center, Danville, USA

**Keywords:** macular hole, lpi, iridotomy, nd:yag, laser

## Abstract

Macular hole formation is a rare complication of laser peripheral iridotomy (LPI) and may cause permanent scotomas. This case describes macular hole formation after 2.8 mJ Nd:YAG laser energy application in a patient with vitreomacular attachment. A literature review of a limited number of case reports shows that older patients who developed holes after LPI had more visually-significant and advanced stage holes that required surgical retinal repair, whereas younger patients had less visually-significant and mild stage holes with a spontaneous resolution on optical coherence tomography (OCT).

## Introduction

Laser peripheral iridotomy (LPI) is a common procedure used by comprehensive and glaucoma ophthalmologists to prevent and treat acute angle-closure glaucoma. However, there may be anterior segment adverse effects including hyphema, iritis, and corneal endothelial damage [1]. In addition, posterior segment complications include malignant glaucoma and retinal detachment [2,3]. Rarely, macular holes have been reported after LPI postulated to be from the concussive force on the anterior hyaloid face propagated within the vitreous cavity [4]. To the authors' knowledge, this case demonstrates the lowest amount of energy reported to cause macular hole formation after LPI. In addition, iatrogenic stage 1A macular holes may cause permanent scotomas.

## Case presentation

**Figure 1:**
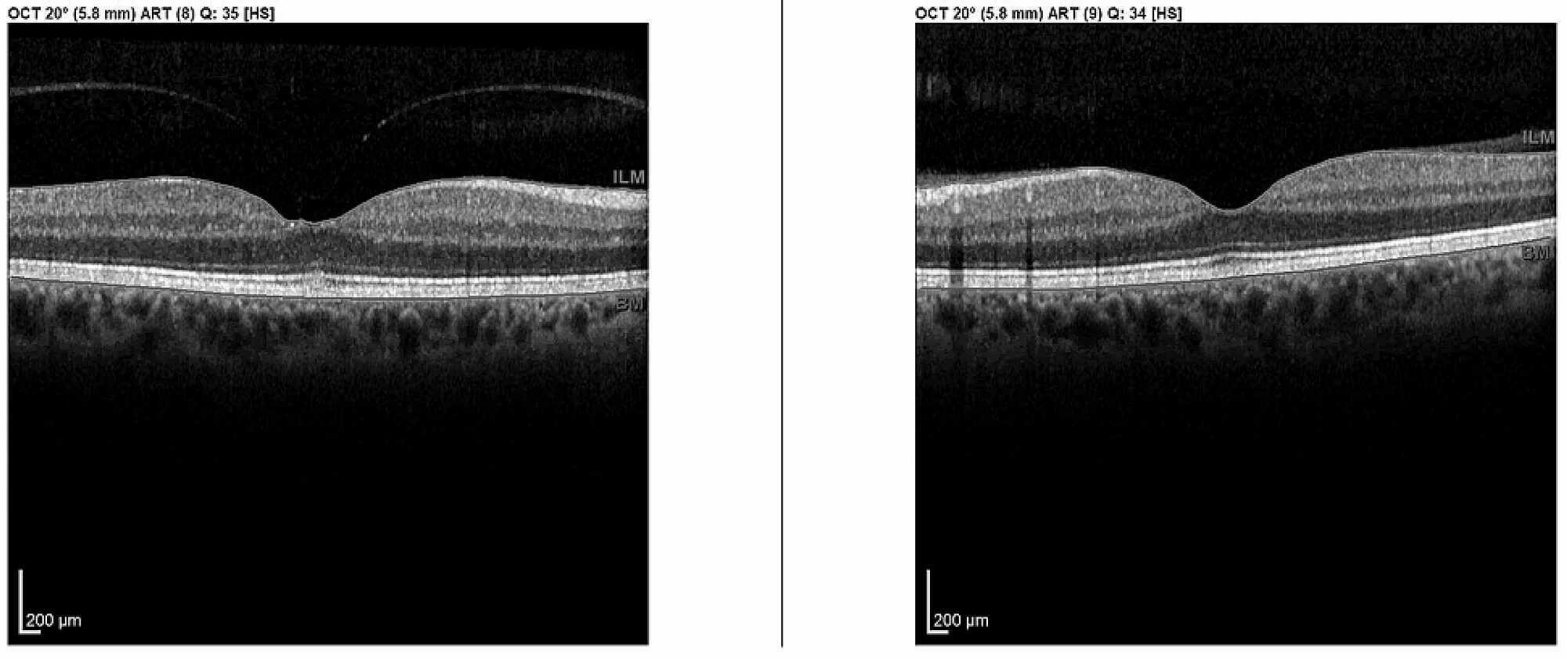
Preprocedural OCT macula horizontal raster demonstrating decreased area of vitreomacular attachment in the right eye compared to the left eye. OCT: optical coherence tomography

A 56-year-old Caucasian male was referred to our department for evaluation of bilateral anatomic narrow angles. Visual acuity was 20/20 in both eyes with an intraocular pressure of 18 mm Hg bilaterally. Gonioscopic examination revealed blue irides with narrow, occludable angles bilaterally. Fundus examination revealed no evidence of optic nerve or vitreomacular pathology. Preoperative spectral-domain optical coherence tomography (OCT) demonstrated mild asymptomatic vitreomacular adhesion in the right eye and vitreomacular attachment in the left eye (Figure 1).

**Figure 2:**
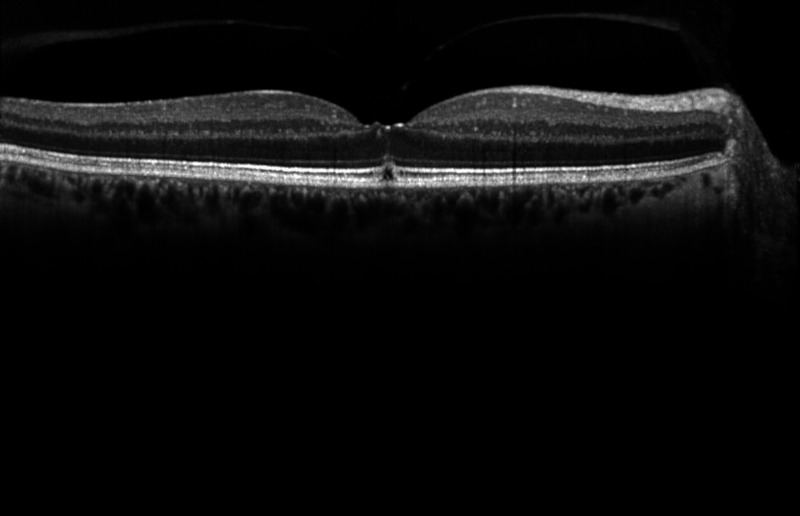
Postprocedural OCT macula horizontal raster of the right eye demonstrating a stage 1A macular hole. OCT: optical coherence tomography

We proceeded with a laser peripheral iridotomy (LPI) of the right eye using Nd:YAG laser requiring two shots and total energy of 2.8 mJ. A patent superior iridotomy was confirmed at the slit-lamp. Eleven days later, the patient presented with abnormal visual disturbance of the right eye noting a dark spot in the center of his vision when reading. Visual acuity was 20/25 in the affected eye and fundus examination revealed a yellow foveolar spot. OCT demonstrated a new stage 1A macular hole of the right eye (Figure 2).

**Figure 3:**
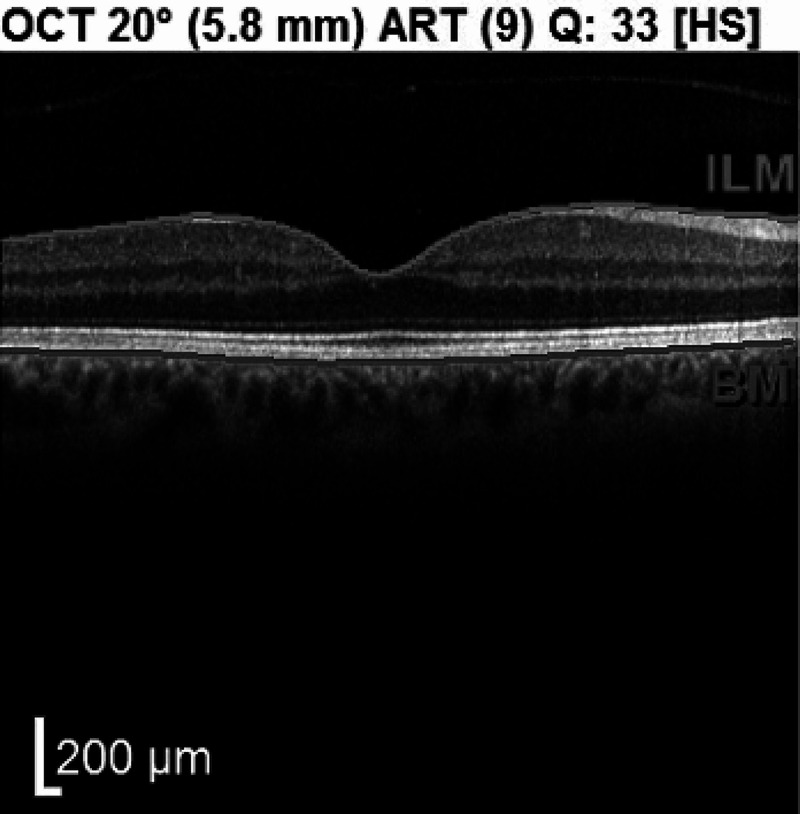
Post-observational OCT macula horizontal raster of the right eye demonstrating anatomic resolution of the stage 1A macular hole and a posterior vitreous detachment. OCT: optical coherence tomography

At four-week follow-up, the patient did not note any improvement in his central scotoma although OCT revealed resolution of the vitreomacular traction (Figure 3).

Six weeks later, the patient presented for laser peripheral iridotomy of the left eye. A total of two shots requiring 3.2 mJ of energy was required to create a patent superior iridotomy. Upon routine follow-up, the macular examination remained normal in the left eye. The patient continued to experience a central scotoma in his right eye. 

## Discussion

The mechanism of macular hole formation after LPI has been postulated to be secondary to plasma formation and photodisruption caused by Nd:YAG force extending into the vitreous cavity. This patient demonstrated macular hole formation after LPI in his right eye and no hole formation after LPI in his left eye although similar energy and technique were utilized bilaterally. This asymmetric result after similar procedures was most likely the result of varying preprocedural levels of vitreomacular attachment in each eye (Figure 1).

In the patient’s right eye there was a decreased area of vitreomacular attachment compared to the left eye. Although similar Nd:YAG force was propagated posteriorly, the smaller vitreomacular interface in the right eye caused an increased level of pressure on the retina compared to the left eye. The left eye had a greater posterior hyaloid attachment and the force utilized, although technically 0.4 mJ higher in the left eye, is believed to be distributed to a larger area of vitreomacular attachment which translated to greater force dispersion and no resultant hole formation.

Syed et al found that increasing age is correlated with an increasing shift in the percentage of patients with partial vitreous separation to a higher proportion of complete posterior vitreous detachment (PVD) [5]. In the 50-54 age group, 71.2% were found to have partial vitreous separation and 1.5% were found to have a complete PVD. In contrast, in the 65-69 age group, only 44.7% were found to have partial vitreous separation and 22.6% had a complete PVD. This reflects vitreous syneresis that occurs with age.

In a review of the literature, one other eye has been reported to have developed a stage 1 macular hole after LPI. In addition, there have been three eyes from two patients who developed stage 4 macular holes [4,6,7]. In this small sample of this rare complication, those who developed stage 1 macular holes were younger (54 and 60), whereas those who developed stage 4 macular holes were older (64 and 69) [4,6,7]. This suggests that the development of advanced macular holes after LPI increases with age in patients who have not yet developed a posterior vitreous detachment, possibly due to decreased area of the vitreomacular interface. Nonetheless, although older patients had a worse postprocedural visual acuity consistent with stage 4 macular holes, these patients experienced an improvement in visual acuity postvitrectomy (Table 1).

**Table 1:** A summary of reported macular hole complications after laser peripheral iridotomy. Follow-up visual acuity is post-observational for stage 1 macular holes and post-vitrectomy for stage 4 macular holes. LPI: laser peripheral iridotomy; OS: oculus sinister; OD: oculus dextrus

Author	Age	Eye	LPI Power (mJ)	Hole Stage	Initial Va	Post-LPI Va	Follow-up Va
Current study	56	OD	2.8	1	20/20	20/25	20/25
Anderson et al., 2006 [4]	60	OS	49.2	1	20/20	20/30	20/20
Sar et al., 2015 [7]	64	OS	5.6	4	6/9	6/36	6/9
Acharya et al., 2008 [6]	69	OD	70	4	6/6	6/24	6/12
		OS	52.8	4	6/6	6/60	6/9

## Conclusions

This case supports the literature in the development of macular hole formation as a side effect after LPI and the potential of developing a permanent visual scotoma. In addition, it highlights that preprocedural OCT evaluation of vitreomacular anatomy may help guide the counseling of posterior segment complications prior to LPI. Furthermore, the case highlights the value of a follow-up dilated fundus examination. A review of this rare complication in this small sample demonstrates that older patients who developed holes after LPI were more visually-significant and advanced stage holes that required surgical retinal repair, whereas younger patients experienced less visually significant and early-stage holes with a spontaneous resolution on OCT.
